# They are more beautiful than me! How social media use increases women’s body-related envy and cosmetic surgery consideration

**DOI:** 10.3389/fpsyg.2025.1628208

**Published:** 2025-08-21

**Authors:** Minhui Li, Xingming Li, Feng Yang, Tianning Zhang

**Affiliations:** ^1^Chongqing University of Technology, Chongqing, China; ^2^Department of Teacher Education, Taishan University, Tai’an, China

**Keywords:** social media, TikTok, body-related envy, social comparison, cosmetic surgery

## Abstract

**Background:**

Past research has indicated the close connection between social media use and women’s envy, but so far, no research has been conducted to exclusively examine the effect and mechanism of social media use on women’s body-related envy.

**Objective:**

To fill this gap, with TikTok as a representative example of video-based social media, four studies (*N* = 767) were conducted to explore whether and how social media use increases women’s body-related envy, and the subsequent downstream consequences.

**Method and results:**

In Study 1a, we employed an online questionnaire survey and found that TikTok use had a significantly positive prediction on women’s body-related envy. In Study 1b, we temporarily activated the TikTok use state in the lab and found that participants in the TikTok use priming condition reported greater body-related envy than those in the control condition. In Study 2, we divided TikTok use into active and passive TikTok use and then applied an online questionnaire survey to examine the relationship and mechanism between active/passive TikTok use and women’s body-related envy. The results showed that passive TikTok use, rather than active TikTok use, had a significant prediction on women’s body-related envy, and appearance upward comparison played a fully mediating role between them. In Study 3, we conducted an online experiment to explore the downstream consequences related to women’s body-related envy. The results showed that, partially *via* the mediating role of body-related envy, passive TikTok use further increased women’s cosmetic surgery consideration.

**Conclusion:**

Passive social media use can significantly increase women’s body-related envy *via* the mediating role of appearance upward comparison. And the increased body-related envy will further increase women’s cosmetic surgery. The present research contributes to understanding how social media use increases women’s body image concerns and appearance enhancement intentions.

## 1 Introduction

There are 1,000 of beautiful women in the world, but half of them only live in TikTok (named Douyin in China-Mainland).

—A humorous remark from Chinese netizens.

By the end of 2023, TikTok had more than 1 billion users worldwide, spanning across all age groups (QuestMobile, 2024). On TikTok, you can always view countless beautiful women who often have a pretty face, a perfect body shape, or both. For the purpose of attracting more users and increasing users’ engagement, TikTok provides various free filters to help users display an ideal body. Similar to TikTok, most picture/video-based social media platforms (e.g., Facebook, Instagram) encourage users to present an ideal body, which significantly increases women’s body image concerns, especially for young women (Modica, 2019; Prichard et al., 2018).

A negative consequence of frequent exposure to ideal bodies on social media is to experience upward social comparison, which refers to compare oneself with a reference target who is better than the self (Festinger, 1954). As those picture/video-based social media platforms often make ideal beauty salient, appearance comparison can be a key dimension of social comparison for social media users (e.g., Fardouly et al., 2015; Lewallen and Behm-Morawitz, 2016; Pan et al., 2023). This is especially true for female users. Past research suggests that, due to appearance upward comparison, social media use is associated with a series of negative body-relevant affective and behavioral consequences for women (Jarman et al., 2021; Seekis et al., 2020; Wang et al., 2020). For example, by conducting an online questionnaire survey, Seekis et al. (2020) found that engaging in appearance-relevant activities on social media was positively linked to women’s appearance anxiety *via* the upward social comparison (Seekis et al., 2020). A similar results pattern was found in a lab study, which showed that participants’ body shame and appearance anxiety were both significantly increased after they were exposed to idealized images (Monro and Huon, 2005). Additionally, social media use is also related to lower body esteem of women and greater anxiety (e.g., Modica, 2019; Yang et al., 2020). Although past research has revealed various body-related consequences related to social media use, surprisingly, little attention has been paid to explore the effect of social media use on appearance envy of women, a commonly negative self-conscious emotion like shame or guilt (Tangney and Salovey, 2010). Considering that appearance upward comparison is common on social media and upward social comparison plays a key role in the generation of envy (Tylka et al., 2023; van de Ven and Zeelenberg, 2020), it is reasonable to expect that social media use contributes to body-related envy of women *via* the mediation of appearance upward comparison. However, so far, there is no direct evidence to support this hypothesized model. To fill this gap, the present research aimed to exclusively examine the effect and mechanism of social media use on female body-related envy.

Noteworthy, an extensive line of research suggests that psychological consequences of social media use can vary with active/passive social media use (e.g., Pan et al., 2023; Quiroz and Mickelson, 2021; Thorisdottir et al., 2019). Passive social media use means that one only browses online content without interacting with others; in contrast, active social media use means that one not only browses the content shared by others but also publishes their own works and is willing to discuss them with other online users (Verduyn et al., 2020). Compared to positive social media use, passive social media use seems to be more likely to make users experience upward social comparison, thus resulting in negative psychological consequences (e.g., Burnell et al., 2019; Hu and Liu, 2020; Yue et al., 2022; but some controversies about this issue, see Valkenburg et al., 2022). For example, with a large sample consisting of adult female TikTok users, Pan et al. (2023) found that passive TikTok use was negatively associated with participants’ appearance- and weight-esteem, but the reversed pattern was obtained for active social media use. Based on prior literature, we expected that compared to active social media use, passive social media use is more likely to induce women’s body-related envy.

Past research demonstrates that body-related envy motivates women to pursue cosmetic surgery to improve their physical appearance (Arnocky et al., 2016). In China, with the development of its economy, more and more women are willing to undergo cosmetic surgery. The number of cosmetic surgery operations in China has accounted for 12.7% of the total number across the world (China National Radio, 2019). However, so far, little attention has been paid to identify possible psychological factors that motivate Chinese women to pursue cosmetic surgery. Given that, the present research attempted to explore whether body-related envy induced by social media use further motivates women to consider cosmetic surgery.

Taken together, with TikTok as a representative example of video-based social media, the present research examined the effect and mechanism of passive/active TikTok use on female body-related envy and the downstream consequences (cosmetic surgery consideration). By doing so, the present research will deepen our understanding of how social media use shapes the self-concept of women and also provides a novel insight into the prevalence of cosmetic surgery in today’s society.

## 2 Literature review

### 2.1 Social comparison on social media

Benefiting from the Web 2.0 technique and user-based generation idea, social media has experienced explosive growth in the past two decades. Compared to traditional mass media, a typical characteristic of social media is to allow users to create their own content on the platform and interact with other online users (Taprial and Kanwar, 2012). For those picture/video-based platforms, as mentioned in the beginning section, they often encourage female users to display ideal bodies to attract the attention of others. Indeed, around the world, those highly popular social media platforms are mostly picture/video-based, such as Facebook or TikTok (Boursier et al., 2020). Additionally, motivated by the purpose of impression management, most women are willing to display an ideal body on social media platforms, even when these depictions significantly deviate from their true selves (Goffman, 1959; McComb and Mills, 2022). As a consequence, the more frequently women use social media, the more likely they are to experience upward social comparisons regarding physical appearance (e.g., Fardouly and Vartanian, 2015; Seekis et al., 2020).

The term “upward social comparison” originates from social comparison theory, which postulates that people have an inner inclination to compare themselves with similar others in a given dimension (e.g., ability, trait), so that they can make quick self-evaluation in a social interaction context (Festinger, 1954). According to the comparison direction, social comparison is categorized into upward and downward social comparisons. Upward comparison occurs when the target is perceived as superior to the self in a given dimension, whereas downward comparison occurs when the target is perceived as inferior to the self. Past research on social media suggests that upward social comparison is typically associated with negative psychological outcomes, such as increased anxiety (Yang et al., 2023), lower self-esteem (Midgley et al., 2021), and greater depression (Alfasi, 2019). Additionally, social media use is also found to correlate significantly with envy (Charoensukmongkol, 2018; Sharifian et al., 2022), a key outcome variable of the present research.

### 2.2 Social media use and body-related envy

Envy is generally defined as an unpleasant feeling that generates when individuals realize that others have something they are longing for but fail to possess (Smith and Kim, 2007). Body-related envy is a specific kind of envy that often emerges when one makes upward social comparison with another whose physical attributes are perceived as superior (Lucibello et al., 2022). Body-related envy is a conceptual construct similar to but distinct from appearance anxiety. Appearance anxiety is characterized by excessive fear of negative evaluation regarding one’s own looks (Hart et al., 1989; Levinson and Rodebaugh, 2012). While body-related envy is driven by appearance upward comparison and longing for others’ attributes (Pila et al., 2014; van de Ven and Zeelenberg, 2020), appearance anxiety often stems from internalized fear of appearance evaluation (Levinson and Rodebaugh, 2012). Additionally, body-related envy is also different from body shame. Body shame is a self-conscious emotion marked by global negative self-evaluation, where individuals feel inherently flawed or defective due to their appearance (Gilbert, 2002; McKinley and Hyde, 1996). At the operational level, the scales designed to assess body-related envy do not measure appearance anxiety or body shame (e.g., Lucibello et al., 2022; Pila, 2013).

Generally, upward social comparison is considered a key factor in the generation of envy (van de Ven and Zeelenberg, 2020). On those picture/video-based platforms, female users often experience appearance upward comparison due to the unrealistic ideal beauty standards promoted on these platforms (Fardouly and Vartanian, 2015; McComb and Mills, 2022). Thus, more frequent social media use in theory will make female users experience more appearance upward comparisons, which further induces female body-related envy. This hypothesis can also be interpreted in the framework of the objectification theory, which posits that women in Western society are given the message that their body is a primary source of their value and worth (Fredrickson and Roberts, 1997; Liss and Erchull, 2015). Women in society tend to treat their bodies as objects and evaluate themselves on the basis of appearance, which is termed as female self-objectification. Today, female self-objectification is a common phenomenon around the world (Koval et al., 2019), and social media use is identified as a key predictor of the phenomenon (Tylka et al., 2023). On social media platforms, women are frequently exposed to appearance-relevant content, resulting in internalizing those beauty ideals. However, due to the unattainability of the so-called ideal beauty standards, women with frequent social media use often experience negative self-conscious emotions, such as body shame (Noll and Fredrickson, 1998), appearance anxiety (Monro and Huon, 2005), and envy (Wang et al., 2020).

It should be pointed out that, although several studies indicate a link between social media use and envy, no research has been conducted to directly test the relationship between social media use and female body-related envy. For example, by adopting a questionnaire survey, Charoensukmongkol (2018) found that there was a significant positive relationship between social media use intensity and envy when their parents compared children and teenagers to their peers. However, Charoensukmongkol (2018) measured envy as a general unpleasant emotion, rather than specifically focusing on body-related envy. Similarly, several other studies that demonstrate the correlation between social comparison on social media and envy do not exclusively focus on body-related envy (Lim and Yang, 2015; Sharifian et al., 2022; Wang et al., 2020). Thus, it remains an open question whether social media use will contribute to female body-related envy. According to previous reasoning, we hypothesized that,

*H1*: In general, social media use significantly and positively predicts female body-related envy.

Noteworthy, social media use is not necessarily linked to negative psychological consequences, as these effects can vary depending on a range of individual and situational factors (Burrow and Rainone, 2017; Modica, 2019; Pan et al., 2023; Rae and Lonborg, 2015). As we have discussed, compared to active social media use, passive social media use is more likely to produce negative psychological consequences. Three possible reasons can explain this phenomenon. First, compared to negative social media use, positive social media use implies that users often publish their own work on social media platforms, which can receive compliments and positive comments from other users (Arroyo and Brunner, 2016). In this case, users may experience downward, rather than upward social comparison, and the former is more likely to result in positive psychological outcomes (Suls et al., 2002; Wills, 1981). Second, when actively interacting with other users, users will perceive social support from others (Carr et al., 2016; Lin et al., 2022). Past research has demonstrated that perceived social support can function as a buffer to largely counteract the negative psychological consequences of upward social comparison (Halbesleben and Buckley, 2006; Han and Yang, 2023). Third, in practice, the more frequently users publish their work on social media, the more likely they realize how the platform operates (e.g., knowing some tips about how to beautify the photos presented on social media), which can help users alleviate the negative psychological consequences of social media use (Vendemia and DeAndrea, 2018; Wang et al., 2024). Supporting this proposition, Vendemia and DeAndrea (2018) found that when women believed that the selfies on Instagram were digitally modified or altered, they were less likely to internalize those photos as beauty standards (Vendemia and DeAndrea, 2018). Given the above considerations, we hypothesized that,

*H2a*: Active social media use does not significantly predict female body-related envy.

*H2b*: Passive social media use significantly and positively predicts female body-related envy.

*H2c*: Appearance upward comparison plays a mediating role between passive social media use and female body-related envy.

### 2.3 Social media use, body-related envy and cosmetic surgery consideration

In the present research, we attempted to explore whether body-related envy induced by social media use would motivate women to consider cosmetic surgery. The evolutionary psychology perspective on envy posits that body-related envy can be regarded as an adaptive emotional response in domains of reproductive relevance (Arnocky et al., 2016; Hill and Buss, 2008). Specifically, compared to women, men place more weight on physical attractiveness in their mates (Buss, 1989; Li et al., 2002). That means, for women, an attractive appearance will effectively increase the probability of finding a high-quality mate. In this sense, when a woman perceives other women as more attractive than herself, it is rational for her to perceive heightened threats from intrasexual rivals and generate a negative emotional response—body-related envy. Importantly, body-related envy can motivate women to take complementary measures to enhance their physical attractiveness, including but not limited to purchasing appearance enhancement products, engaging in risky weight-loss behaviors, and pursuing cosmetic surgery (Arnocky et al., 2012, 2016; Nabi and Keblusek, 2014; Pila et al., 2014). Applying a similar logic, the objectification theory can also be used to explain why social media use contributes to women’s pursuit of cosmetic surgery (Fredrickson and Roberts, 1997). Due to exposure to unrealistic ideal bodies on social media, female social media users frequently experience upward social comparison in the domain of physical appearance (Seekis et al., 2020; Yang et al., 2020). As a consequence, more frequent social media use generates more negative emotions related to physical appearance, such as body shame and body dissatisfaction (Manago et al., 2015; Perloff, 2014). To alleviate such negative emotions, women tend to engage in some complementary activities (Calogero et al., 2010, 2014; Melbye et al., 2007; Noll and Fredrickson, 1998). For example, by using a scrambled sentence task to prime female undergraduates’ self-objectification state, Calogero et al. (2014) found that self-objectification increased participants’ body shame and intentions to have cosmetic surgery, and body shame partially mediated the relationship between self-objectification and cosmetic surgery consideration. Overall, both the evolutionary psychology perspective of envy and the objectification theory, more or less, indicate the possibility that social media use will motivate women to pursue cosmetic surgery *via* the role of body-related envy.

As we have mentioned, social media use can be classified into active and passive social media use. Although both active and passive social media use has been found to correlate with higher intentions to have cosmetic procedures, different mechanisms may underlie these relationships (Hermans et al., 2022). According to our previous reasoning, passive (but not active) social media use should contribute to female body-related envy. Thus, we speculated that passive social media use may increase women’s cosmetic surgery consideration *via* the mediating role of body-related envy. For active social media use, we speculated that internalization of beauty ideals may be a possible mediation mechanism between active social media use and women’s cosmetic surgery consideration. For example, recent research by Wang et al. (2024) found that exposure to selfies on social media was significantly and positively correlated with adolescents’ internalization of beauty ideals, and internalization of beauty ideals was found to have a significant and positive correlation with adolescents’ cosmetic surgery consideration. However, considering that female body-related envy was a key variable of the present research, we thus exclusively examined whether passive social media use motivates women to consider cosmetic surgery *via* the mediating role of body-related envy. And we did not pay additional attention to investigate how active social media use increases women’s cosmetic surgery consideration in the present research. Our hypotheses are as follows:

*H3a*: Passive social media use significantly and positively predicts women’s cosmetic surgery consideration.

*H3b*: Body-related envy plays a mediating role between passive social media use and women’s cosmetic surgery consideration.

## 3 The present research

In the present research, with TikTok as a representative example of video-based social media, we conducted four studies to explore whether and how social media use contributes to female body-related envy and the subsequent downstream consequences. In Study 1a, we employed an online questionnaire survey to examine the relationship between TikTok use and female body-related envy. In Study 1b, we temporarily activated the TikTok use state in the lab, and then examined the effect of TikTok use on female body-related envy. In Study 2, we distinguished between active and passive TikTok use, and then applied an online questionnaire survey to examine the relationship between active/passive TikTok use and female body-related envy, and the mediating role of appearance upward comparison between them. In Study 3, we conducted an online experiment to explore the downstream consequences related to the effect of passive TikTok use on female body-related envy. Specifically, we would examine whether passive TikTok use produces an influence on women’s cosmetic surgery consideration *via* the mediation of body-related envy. Summaries of the four studies are presented in Table 1.

**Table 1:** Summaries of the four studies.

Studies	Participants	Objectives	Design	Data analyses	Hypotheses
Study 1a	260 adult women	To examine the relationship between SMU and female BE	An online questionnaire survey	A hierarchical regression analysis	SMU has a significant prediction on female BE (H1).
Study 1b	110 female undergraduates	To examine the effect of SMU on female BE.	A lab experiment	An independent-samples *t*-test	Participants in the SMU condition show greater BE compared to those in the control condition (H1).
Study 2	200 adult women	To examine the relationship and mechanism between active/passive SMU and female BE	An online questionnaire survey	Hierarchical regression analyses; A mediation analysis for appearance upward comparison	Positive SMU does not have a significant prediction on female BE (H2a); Negative SMU has a significant prediction on female BE (H2b); Appearance upward comparison plays a mediating role between negative SMU and female BE (H2c).
Study 3	200 adult women	To explore the downstream consequences related to the effect of SMU on female BE	An online experiment	An independent-samples *t*-test; A mediation analysis for female BE	Participants in the negative SMU condition show more cosmetic surgery consideration than those in the control condition (H3a); Negative SMU produces an influence on female cosmetic surgery consideration *via* the mediation of BE (H3b).

SMU, social media use; BE, body-related envy. The sample in each study is independent of the others.

## 4 Study 1a

Study 1a was an online questionnaire survey, whose goal was to examine whether TikTok use can significantly predict female body-related envy.

### 4.1 Methods

#### 4.1.1 Participants

Following the recommendations of Cheng et al. (2024a,b), we use the Monte Carlo simulations developed by Schönbrodt and Perugini (2013) to determine the sample size. According to the calculation of the Monte Carlo simulation, approximately 250 participants are needed to obtain a reliable estimation of bivariate correlations. Considering possible invalid data, we recruited 260 women as participants on the Credamo platform,^1^ which is a professional data collection platform similar to MTurk. In addition to the gender restriction (self-identified as female), a qualified participant must have at least one TikTok account. Due to unrealistically short response times, three participants were excluded from any data analysis. As a result, a total of 257 participants were included in the final data analysis.

The average age of participants was 31.51 years old (*SD* = 7.97), fluctuating from 17.75 to 60.00 years old. Past research demonstrates that BMI plays a critical role in shaping women’s body image concerns (e.g., Cella et al., 2020). We thus calculated participants’ BMI by dividing one’s weight (kg) by her height (meter) squared. This calculation generated a mean value *M* = 20.99 (*SD* = 2.77), with a fluctuation range of 13.30 ~ 36.73. A total of 226 participants lived in the city, and the remaining 31 participants lived in the country. The majority of participants were of Han nationality (95.72%), and the remaining participants were national minorities. The proportion of participants with a bachelor’s degree or above was 89.90%.

#### 4.1.2 Measures

##### 4.1.2.1 TikTok use

Following previous research (Olson et al., 2012), a six-item scale that was derived from the research by Ellison et al. (2007) was used to assess participants’ TikTok use intensity. The original scale was developed to measure Facebook use intensity, and its Chinese version has shown good psychometric properties (Qiu et al., 2017). In the study, we replaced the term “Facebook” in the scale with “TikTok,” such as “TikTok has become part of my daily routine.” For each item, participants needed to give their agreement on the 6-point scale (0 = *strongly disagree*, 5 = *strongly agree*). TikTok use intensity was assessed by summing participants’ scores on each item, with a higher score indicating greater use intensity. In this study, the Cronbach’s *α* of the TikTok Use Intensity Scale was 0.88.

##### 4.1.2.2 Body-related envy

Body-related envy was measured by the Body-related Envy Scale provided by Pila (2013). Through guiding individuals to focus on the physical self, Pila (2013) modified the Dispositional Envy Scale developed by Smith et al. (1999) into the Body-related Envy Scale. An example item is “It is so frustrating to see some people who have great bodies/physiques with little effort.” The modified scale contained eight items, and participants needed to provide their agreement on the 5-point scale (1 = *strongly disagree*, 5 = *strongly agree*). Given that there was no available Chinese version of the Body-related Envy Scale, the back-translation procedure developed by Brislin (1980) was employed to ensure equivalence between the Chinese and English versions. The confirmatory factor analysis (CFA) demonstrated good model fit indices: *χ*^2^/df = 2.16, RMSEA (Root Mean Square Error of Approximation) = 0.07, GFI (Goodness of Fit Index) = 0.98, TLI (Tucker-Lewis Index) = 0.97, SRMR (Standardized Root Mean Square Residual) = 0.03. Body-related envy was assessed by averaging the score of all items, with a higher value indicating greater body-related envy. In this study, the Cronbach’s α of the Body-related Envy Scale was 0.93.

##### 4.1.2.3 Dispositional envy in general

Following previous research (Tandoc and Goh, 2023), three items were used to measure dispositional envy of participants: (1) I generally feel inferior to others, (2) many of my friends have a better life than me, and (3) many of my friends are happier than me. For each item, participants needed to give their agreement on the 5-point scale (1 = *strongly disagree*, 5 = *strongly agree*). Due to the lack of an available Chinese version of the dispositional scale, Brislin (1980) back-translation procedure was again used to achieve linguistic equivalence between the translated Chinese version and the original English scale. Considering that the scale has only three items, we did not perform CFA. Dispositional envy was calculated by averaging the score of all items, with a higher value indicating greater dispositional envy. In this study, the Cronbach’s α of the Dispositional Envy Scale was 0.88.

##### 4.1.2.4 Demographic information

In Study 1a, the collected demographic information included age, nationality, height, weight, education degree (1 = *primary school*, 2 = *junior high school*, 3 = *senior high school*, 4 = *junior college*, 5 = *bachelor*, 6 = *master*, 7 = *doctor*), and family monthly income (1 = *less than 3,000 YUAN*, 2 = *3,000 ~ 5,000 YUAN*, 3 = *5,000 ~ 8,000 YUAN*, 4 = *8,000 ~ 12,000 YUAN*, 5 = *12,000 ~ 15,000 YUAN*, 6 = *15,000 ~ 20,000 YUAN*, 7 = *more than 20,000 YUAN*).

#### 4.1.3 Procedure

Study 1a was administered on the Credamo platform. Only eligible women were allowed to participate in this survey (see *Participants* section). At the beginning of the survey, we told participants that this survey was about TikTok use and female self-perception. If they were willing to continue with the survey, they needed to sign the electronic informed consent. After that, they successively completed each part of the survey and then submitted it. In return, each participant received 2 YUAN (approximately 0.3 USD).

### 4.2 Results

#### 4.2.1 Descriptive statistical results

The questionnaire could be successfully submitted only if all items were completed. So, no missing values were generated in Study 1a. Data analysis was performed using SPSS 26.0. Correlations among variables were presented in Table 2. As shown in the table, TikTok use was positively and significantly correlated with body-related envy, *r* = 0.13, *p* < 0.05, and TikTok use was also positively and significantly correlated with family monthly income, *r* = 0.26, *p* < 0.001. Body-related envy was positively and significantly correlated with dispositional envy, *r* = 0.76, *p* < 0.001, and BMI, *r* = 0.18, *p* < 0.01. Additionally, body-related envy was negatively and significantly correlated with family monthly income, *r* = −0.13, *p* < 0.05. For a concise presentation, those significant correlations irrelevant to TikTok use or body-related envy were presented only in Table 2.

**Table 2:** Means, standard deviations, and correlations among variables in Study 1a.

Variables	*M*	SD	TikTok use	Body-related envy	Dispositional envy	BMI	Age	Education degree	Family monthly income
TikTok use	23.39	5.18	1						
Body-related envy	2.86	1.00	0.13^*^	1					
Dispositional envy	2.58	1.03	0.001	0.76^***^	1				
BMI	20.99	2.77	−0.02	0.18^**^	0.16^*^	1			
Age	31.51	7.97	0.10	−0.07	−0.04	0.20^**^	1		
Education degree	5.02	0.60	−0.08	−0.05	−0.10	−0.04	−0.04	1	
Family monthly income	3.52	1.47	0.26^***^	−0.13^*^	−0.17^**^	0.11	0.42^***^	0.13^*^	1

M, mean; SD, standard deviation; BMI, body mass index. ^*^p < 0.05, ^**^p < 0.01, ^***^p < 0.001.

#### 4.2.2 The relationship between TikTok use and body-related envy

Preliminary analyses confirmed that the data met all assumptions of linear regression, including linearity, normality of residuals, and homoscedasticity. The Tolerance and Variance Inflation Factor (VIF) values were very close to 1 for all predictors (Tolerance = 0.90–0.97; VIF = 1.00–1.11), indicating no significant multicollinearity (Belsley et al., 2005). Next, we conducted a hierarchical regression equation to examine whether TikTok use would have a significant prediction effect on female body-related envy. We determined which variables to include in the regression equation based on the results of the correlation analyses. In the first step, dispositional envy was entered into the equation in the first step, and BMI and family monthly income were entered into the equation in the second step. Finally, the TikTok use was entered into the equation. All variables were standardized before they were entered into the equation. As shown in Table 3, when the effects of all control variables were taken into account, TikTok use still had a significantly positive prediction effect on female body-related envy, *β* = 0.14, *t* = 3.48, *p* = 0.001, 95% CI [0.06, 0.22].

**Table 3:** The hierarchical regression analysis results in Study 1a.

Regression model	Outcome variable	Prediction variable	Model summary			Standardized regression coefficients			
			*F*	*p*	*R* ^2^	*β*	*t*	*p*	95% CI
Model 1	Body-related envy	Dispositional envy	346.11	< 0.001	0.58	0.76	18.60	< 0.001	[0.69, 0.83]
Model 2	Body-related envy	Dispositional envy	116.08	< 0.001	0.58	0.75	17.86	< 0.001	[0.68, 0.82]
		BMI				0.06	1.43	0.15	[−0.02, 0.14]
		Family monthly income				−0.002	−0.04	0.97	[−0.13, 0.06]
Model 3	Body-related envy	Dispositional envy	93.91	< 0.001	0.60	0.74	18.04	< 0.001	[0.67, 0.81]
		BMI				0.06	1.58	0.12	[−0.01, 0.15]
		Family monthly income				−0.04	−0.95	0.34	[−0.13, 0.06]
		TikTok use				0.14	3.48	0.001	[0.06, 0.22]

BMI, body mass index.

### 4.3 Discussion

By conducting a questionnaire survey, Study 1a examined the relationship between TikTok use and female body-related envy. The results showed that, consistent with H1, after considering the influences of possible confounding variables, TikTok use had a significantly positive prediction on female body-related envy. However, Study 1a was a correlational design, which did not allow us to draw a causal inference between TikTok use and female body-related envy. Thus, Study 1a provided only preliminary evidence for H1. In the next study, we will conduct a lab experiment to provide convincing evidence for H1.

## 5 Study 1b

Study 1b was a lab experiment whose goal was to provide causal evidence for the effect of TikTok use on body-related envy. We used the priming technique to activate participants’ TikTok use state and then examined whether any differences existed in female body-related envy between the priming and no-priming conditions. Detailed information about experimental manipulation was provided in the *Materials and Measures* section.

### 5.1 Methods

#### 5.1.1 Participants

The sample size of Study 1b was determined *via* G*power 3.1 (Faul et al., 2009). We noticed that some researchers determined the assumed effect size to be medium when calculating the required sample size using G*Power 3.1 (e.g., Gkinopoulos et al., 2023). According to Cohen (1988) suggestion, a medium effect size in a *t*-test is conventionally defined as *d* = 0.5. Thus, when calculating the sample size using G*Power 3.1, we set the effect size to *d* = 0.5, statistical power (1–β) = 0.8, and the significance level (*α*) to 0.05. Based on this estimation, 110 female undergraduates were recruited to participate in Study 1b. Their average age was 21.26 years old (*SD* = 0.95), ranging from 19.33 to 23.17 years old. Their average BMI was 20.24 (*SD* = 1.92), fluctuating from 16.18 to 26.62. Notably, 96 participants lived in the city and 14 participants lived in the country. A total of 105 participants were of Han nationality, and the others were national minorities (2 Hui, 1 Buyi, 1 Uyghur, and 1 Zhuang).

#### 5.1.2 Materials and measures

##### 5.1.2.1 Experimental manipulation

**Figure 1:**
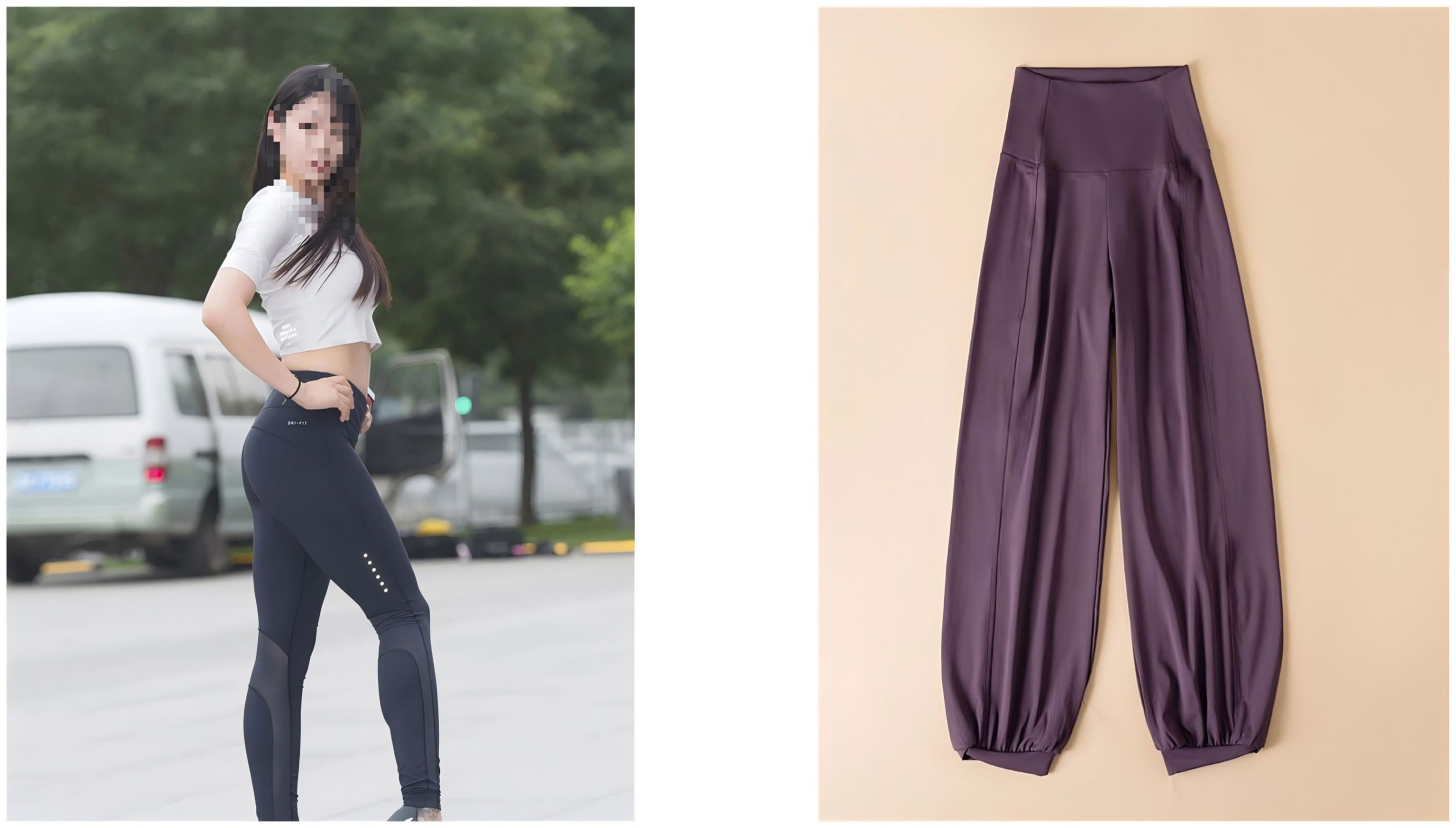
Sample pictures in the TikTok use priming task of Study 1b. The left picture shows an example in the TikTok use priming condition, and the right picture shows an example in the control condition.

Prior literature suggests that exposure to appearance-relevant content on social media can increase women’s body image concerns and appearance upward comparisons (e.g., Fardouly et al., 2015; Sun et al., 2020). Based on previous findings, we primed participants’ TikTok use state *via* pictures that highlight women’s physical appearance (a similar manipulation, see Tiggemann and Zaccardo, 2015; also see Yan et al., 2025). Specifically, in the TikTok use priming condition, we sent a TikTok link to participants *via* the prepared iPads, which introduced the materials, characteristics, wearing suggestions, and suitable scenarios of yoga pants. To make appearance-relevant content salient, 10 pictures of young women in yoga pants were embedded in the text (see Figure 1). Recent research indicates that presenting images of women wearing yoga pants significantly heightens women’s body image concerns (Yan et al., 2025). In the control condition, participants were presented with a PDF document introducing yoga pants. The text in the document was identical to that in the TikTok use priming condition. However, in the control condition, 10 pictures of yoga pants, rather than 10 pictures of women wearing yoga pants, were embedded in the text (see Figure 1). We acknowledge that presenting images of yoga pants is not an “absolutely neutral” condition. The rationale for presenting images of women wearing yoga pants in the experimental condition, while presenting images of yoga pants alone in the control condition, was to rule out one possibility—participants’ body image concerns were heightened by gender-associated symbols linked to women (e.g., yoga pants), rather than appearance-relevant content.

We ostensibly told participants that they needed to carefully browse the content about yoga pants within 5 min because such content would be tested in the subsequent task. To reinforce the experimental manipulation, we explicitly told participants that they should not only pay attention to the text but also pay attention to the pictures. All pictures and text materials were provided on OSF.^2^

##### 5.1.2.2 Body-related envy

Following the TikTok use priming task, participants were asked to report, at this moment, to what extent they felt the following emotions on the 5-point scale (1 = *never*, 5 = *extremely*): resentful, longing for, jealous, and covetous. Body-related envy was assessed by summing the scores of the four emotions (Pila, 2013). To reduce participants’ suspicion of the research purpose, 12 other emotions were added as filler items (Pila, 2013).

#### 5.1.3 Procedure

Participants were recruited from a university in Shandong Province, China. They learned about the recruitment information for the experiment *via* WeChat Moments or WeChat groups. We told participants that they would participate in an experiment about women’s appearance self-perception. When arriving at the lab, they needed to sign the informed consent. Then, half of the participants were assigned to the TikTok use priming condition, and the other half of the participants were assigned to the control condition. It should be pointed out that participants in the TikTok use priming condition were not allowed to post comments about the presented content or engage in online communications. Following the experimental manipulation, participants reported temporary emotions and demographic information on a separate sheet of A4 size. Finally, we debriefed participants, explaining that the study’s true purpose was to investigate how TikTok usage influences women’s body-related envy. Those who expressed interest could receive additional details about the experimental design *via* email. Each participant would receive 5 YUAN (approximately 0.7 USD) as compensation for their participation.

### 5.2 Results

#### 5.2.1 Manipulation effectiveness check

Data analysis was performed using SPSS 26.0. The priming effectiveness of TikTok use was checked by asking participants to what extent the presented materials highlighted appearance-relevant content. They reported their feelings on the 7-point scale (1 = *not at all*, 7 = *strongly*). An independent-samples *t*-test showed that the presented materials in the TikTok use priming condition (*M* = 6.44, *SD* = 0.66) were considered to more strongly highlight appearance-relevant content than those in the control condition (*M* = 3.65, *SD* = 1.81), *t*(108) = 10.72, *p* < 0.001, *d* = 2.04.

#### 5.2.2 The effect of the TikTok use prime on body-related envy

**Figure 2:**
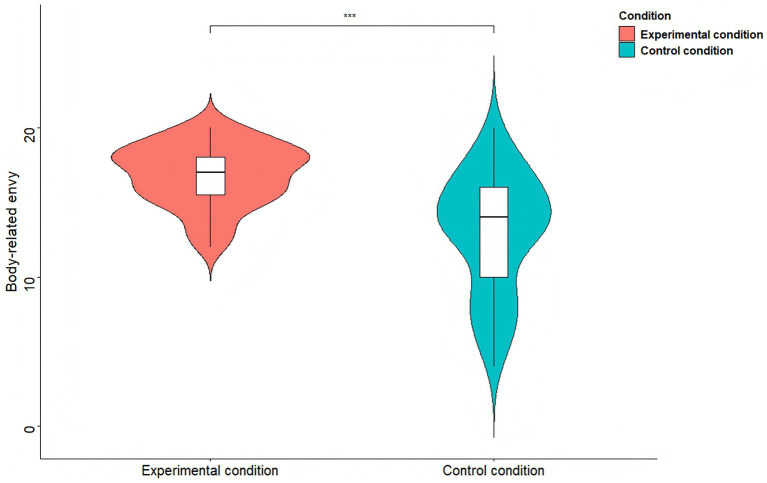
Body-related envy comparisons between the two conditions of Study 1b. ^***^p < 0.001.

An independent-samples *t*-test was conducted to examine whether the TikTok use prime increased women’s body-related envy. The results showed that, as shown in Figure 2, participants in the TikTok use priming condition (*M* = 16.82, *SD* = 2.07) reported greater body-related envy than those in the control condition (*M* = 12.96, *SD* = 3.96), *t*(108) = 6.40, *p* < 0.001, *d* = 0.81.

### 5.3 Discussion

By conducting a lab experiment, Study 1b found that participants in the TikTok use priming condition reported greater body-related envy than those in the control condition, thus providing convincing evidence for H1. It should be pointed out that participants in the TikTok use priming condition were exposed to the appearance-relevant content on TikTok, but they were not allowed to comment on the content or interact with each other. In this sense, the primed TikTok use state in Study 1b should belong to negative TikTok use. In the *Literature Review* section, we have hypothesized that negative TikTok use, but not active TikTok use, will significantly predict female body-related envy. Given that, we would divide TikTok use into active and passive use in the following Study 2, and then examine how they are related to female body-related envy.

## 6 Study 2

Study 2 was an online questionnaire survey. In Study 2, we divided TikTok use into active and passive TikTok use and then examined the relationship between these two types of TikTok use and female body-related envy. We also examined the possible mediating role of appearance upward comparison in the relationship between passive TikTok use and female body-related envy. By doing so, Study 2 would provide evidence for H2a, 2b, and 2c.

### 6.1 Methods

#### 6.1.1 Participants

As in Study 1a, the sample size of Study 2 was determined *via* the Monte Carlo Power Analysis for Indirect Effects application developed by Schoemann et al. (2017). The application showed that a presupposed medium effect size (statistical power 1-*β* = 0.8, correlations among variables *r* = 0.30, *SD* = 0.10) required at least 153 participants. Based on this, a total of 200 women were recruited from the Credamo platform. Their average age was 30.55 years old (*SD* = 7.58), ranging from 18.67 to 57.00 years old. The mean value of participants’ BMI was 20.30 (*SD* = 2.18), ranging from 16.33 to 28.40. A total of 192 participants were of Han nationality, and the others were national minorities (3 Man, 3 Yi, 1 Meng, and 1 Zhuang). Noting that 164 participants lived in the city and 18 participants lived in the country. Of note, 85% of participants reported that they had a bachelor’s degree or above.

#### 6.1.2 Measures

##### 6.1.2.1 Active and passive TikTok use

Active and passive TikTok use was measured *via* the Active and Passive Social Media Use Scale developed by Chen et al. (2022) in Chinese cultural contexts. The scale consists of 10 items. Among them, six items are used to assess active social media use (e.g., posting status updates on social media), and the other four items are used to assess passive social media use (e.g., browsing others’ homepages). In Study 2, after deleting one item of the Passive Social Media Use Subscale (checking to see what others are up to), the Cronbach’s alpha of the subscale increased from the original 0.62–0.78. It should be noted that the original scale did not specify which social media platform(s) participants needed to report their activities on Chen et al. (2022). In this study, we explicitly told participants that they should report their activities on TikTok. Although “checking to see what others are up to” is a common behavior when using social media with strong relational closeness (e.g., WeChat), this behavior is indeed less common on TikTok. Given that, we removed the item “checking to see what others are up to,” even though the reliability coefficient of the Passive Social Media Use Subscale, including this item, would still be acceptable. The reliability coefficient of the Active Social Media Use Subscale was 0.69 in this study.

##### 6.1.2.2 Appearance upward comparison

In Study 2, the five-item Physical Appearance Comparison Scale developed by Thompson et al. (1991) was used to assess participants’ appearance comparison tendency. An example item was “I find myself comparing my appearance with people who are better looking than me.” Participants needed to indicate to what extent they agreed with each item on the 5-point scale (1 = *strongly disagree*, 5 = *strongly agree*). The score of appearance upward comparison was calculated by averaging the scores on all items, with higher scores indicating more appearance upward comparisons. The Chinese version of the Physical Appearance Comparison Scale was developed by Yang et al. (2010) and demonstrated acceptable psychometric properties. In this study, the Cronbach’s *α* of the scale was 0.89.

##### 6.1.2.3 Body-related envy

The measure of body-related envy was identical to that of Study 1a. That is, the modified Dispositional Envy Scale developed by Pila (2013) was used to measure body-related envy. In the study, the Cronbach’s α of the scale was 0.91.

##### 6.1.2.4 Control variables

Prior literature suggests that there is a close connection between envy and self-esteem (Barth, 1988; Vrabel et al., 2018). To reduce the impact of confounding variables as much as possible, we measured participants’ global self-esteem *via* the Chinese version of the Rosenberg Self-esteem Scale (Rosenberg, 1965; Wang et al., 1999). The Rosenberg Self-esteem Scale contains 10 items (e.g., I feel that I am a person of worth, at least on an equal plane with others), and participants needed to provide their agreement for each item on the 4-point scale (1 = *strongly disagree*, 4 = *strongly agree*). The score of global self-esteem was calculated by summing the scores on all items, with higher values indicating greater global self-esteem. As in Study 1a, we also measured participants’ dispositional envy *via* the envy scale developed by Tandoc and Goh (2023). The envy score was calculated by averaging the scores of all items, with a higher value indicating greater dispositional envy.

##### 6.1.2.5 Demographic information

We asked participants to report necessary demographic information, including age, nationality, residence (city/country), family monthly income, education degree, height, and weight.

#### 6.1.3 Procedure

The survey was conducted on the Credamo platform. Only women who had at least one TikTok account were allowed to take part in the survey. Before the formal survey, participants read a brief introduction to the survey. They learned that this was a questionnaire survey about TikTok use and female self-perception. If they were willing to participate in the survey, they needed to sign the electronic informed consent. Then, they successively completed each part of the questionnaire. When they completed the whole questionnaire and submitted it to the platform, they would receive 2 YUAN (approximately 0.3 USD).

### 6.2 Results

#### 6.2.1 Descriptive statistical results

Data analysis was performed using SPSS 26.0. Correlations among variables were presented in Table 4. As shown in the table, appearance upward comparison was significantly and positively correlated with active TikTok use, *r* = 0.17, and passive TikTok use, *r* = 0.27, *p* < 0.01. Passive TikTok use was significantly and positively correlated with body-related envy, *r* = 0.24, *p* < 0.01. Dispositional envy was significantly and negatively correlated with active TikTok use, *r* = −0.15, *p* < 0.05, but significantly and positively correlated with passive TikTok use, *r* = 0.19, *p* < 0.01. Additionally, dispositional envy was significantly and positively correlated with appearance upward comparison, *r* = 0.19, *p* < 0.01, and body-related envy, *r* = 0.54, *p* < 0.001. Global self-esteem was significantly and positively correlated with active TikTok use, *r* = 0.23, *p* < 0.01, but significantly and negatively correlated with body-related envy, *r* = −0.44, *p* < 0.001.

**Table 4:** Correlations among variables in Study 2.

Variables	ATU	PTU	Appearance comparison	Body-related envy	Dispositional envy	Global self-esteem	Age	BMI	Monthly income	Education degree
ATU	1									
PTU	−0.06	1								
Appearance comparison	0.17^*^	0.27^**^	1							
Body-related envy	0.01	0.24^**^	0.41^***^	1						
Dispositional envy	−0.14^*^	0.19^**^	0.19^**^	0.54^***^	1					
Global self-esteem	0.23^**^	−0.15	−0.15	−0.44^***^	−0.75^***^	1				
Age	0.20^**^	−0.13	−0.01	−0.11	−0.24^**^	0.26^***^	1			
BMI	0.02	−0.02	0.02	0.22^**^	0.13	−0.10	0.20^**^	1		
Monthly income	0.24^**^	−0.08	0.07	0.03	0.21^**^	0.20^**^	0.45^***^	−0.09	1	
Education degree	−0.02	−0.10	−0.07	0.01	−0.05	−0.05	−0.03	−0.16^*^	0.29^***^	1

ATU, active TikTok use; PTU, passive TikTok use; BMI, body mass index. ^*^p < 0.05, ^**^p < 0.01, ^***^p < 0.001.

Age was significantly and positively correlated with active TikTok use, *r* = 0.20, *p* < 0.01. Additionally, age was significantly and positively correlated with global self-esteem, *r* = 0.26, *p* < 0.01, but significantly and negatively correlated with dispositional envy, *r* = −0.24, *p* < 0.01. BMI was significantly and positively associated with body-related envy, *r* = 0.22, *p* < 0.01. Family monthly income was significantly and positively correlated with active TikTok use, *r* = 0.24, *p* < 0.01. For a concise presentation, the significant correlation results irrelevant to key variables were only presented in Table 4.

#### 6.2.2 The prediction effect of active/passive TikTok use on body-related envy

To examine whether active/passive TikTok use would have a significant prediction effect on body-related envy, two hierarchical regression equations were conducted separately. Preliminary analyses confirmed that the data satisfied all assumptions of linear regression. The Tolerance and VIF values for all predictor variables in both regression equations fall within acceptable ranges (Tolerance = 0.43–0.96; VIF = 1.08–2.42), indicating no severe multicollinearity in either equation (Belsley et al., 2005). We determined which variables to include in the regression equation based on the results of the correlation analyses. For active social media use, based on the above correlation analysis results, BMI, age, and family monthly income were entered into the equation in the first step. Dispositional envy and global self-esteem were entered into the equation in the second step. Active TikTok use was entered into the equation in the third step. All variables were standardized before they were entered into the equation. The results showed that (see Table 5), after controlling for the effects of control variables, active TikTok use did not show a significant prediction effect on body-related envy, *β* = 0.07, *t* = 1.08, *p* = 0.28, 95% CI (−0.05, 0.18).

**Table 5:** Body-related envy was regressed onto active TikTok use in Study 2.

Regression model	Outcome variable	Prediction variable	Model summary			Standardized regression coefficients			
			*F*	*p*	*R* ^2^	*β*	*t*	*p*	95% CI
Model 1	Body-related envy	Age	6.62	< 0.001	0.09	−0.24	−2.99	0.003	[−0.39, −0.08]
		Monthly income				0.16	2.05	0.04	[0.01, 0.31]
		BMI				0.28	3.95	< 0.001	[0.14, 0.42]
Model 2	Body-related envy	Age	20.48	< 0.001	0.35	−0.11	−1.55	0.12	[−0.24, 0.03]
		Monthly income				0.21	3.14	0.002	[0.08, 0.34]
		BMI				0.19	3.08	0.002	[0.07, 0.31]
		Dispositional envy				0.46	5.12	< 0.001	[0.28, 0.63]
		Global self-esteem				−0.10	−1.07	0.29	[−0.27, 0.08]
Model 3	Body-related envy	Age	17.28	< 0.001	0.35	−0.11	−1.61	0.11	[−0.24, 0.03]
		Monthly income				0.20	2.95	0.004	[0.06, 0.33]
		BMI				0.19	3.04	0.03	[0.07, 0.31]
		Dispositional envy				0.45	5.04	< 0.001	[0.27, 0.63]
		Global self-esteem				−0.11	−1.23	0.22	[−0.29, 0.07]
		Active TikTok use				0.07	1.08	0.28	[−0.05, 0.18]

BMI, body mass index. Standardized regression coefficients were reported.

A similar hierarchical regression equation was conducted to assess the prediction effect of passive TikTok use on body-related envy. As shown in Table 6, after controlling for the effects of control variables, passive TikTok use still showed a significantly positive prediction effect on body-related envy, *β* = 0.16, *t* = 2.63, *p* = 0.009, 95% CI (0.04, 0.27).

**Table 6:** Body-related envy was regressed onto passive TikTok use in Study 2.

Regression model	Outcome variable	Prediction variable	Model summary			Standardized regression coefficients			
			*F*	*p*	*R* ^2^	*β*	*t*	*p*	95% CI
Model 1	Body-related envy	Age	6.62	< 0.001	0.09	−0.24	−2.99	0.003	[−0.39, −0.08]
		Monthly income				0.16	2.05	0.04	[0.01, 0.31]
		BMI				0.28	3.95	< 0.001	[0.14, 0.42]
Model 2	Body-related envy	Age	20.48	< 0.001	0.35	−0.11	−1.55	0.12	[−0.24, 0.23]
		Monthly income				0.21	3.14	0.002	[0.08, 0.34]
		BMI				0.19	3.10	0.002	[0.07, 0.31]
		Dispositional envy				0.46	5.12	< 0.001	[0.28, 0.63]
		Global self-esteem				−0.10	−1.07	0.29	[−0.27, 0.08]
Model 3	Body-related envy	Age	18.75	< 0.001	0.37	−0.10	−1.39	0.17	[−0.23, 0.04]
		Monthly income				0.21	3.19	0.002	[0.08, 0.34]
		BMI				0.19	3.20	0.002	[0.07, 0.31]
		Dispositional envy				0.43	4.85	< 0.001	[0.26, 0.60]
		Global self-esteem				−0.10	−1.08	0.28	[−0.27, 0.08]
		Passive TikTok use				0.15	2.63	0.009	[0.04, 0.27]

BMI, body mass index. Standardized regression coefficients were reported.

#### 6.2.3 The mediating role of appearance upward comparison between passive TikTok use and body-related envy

**Figure 3:**
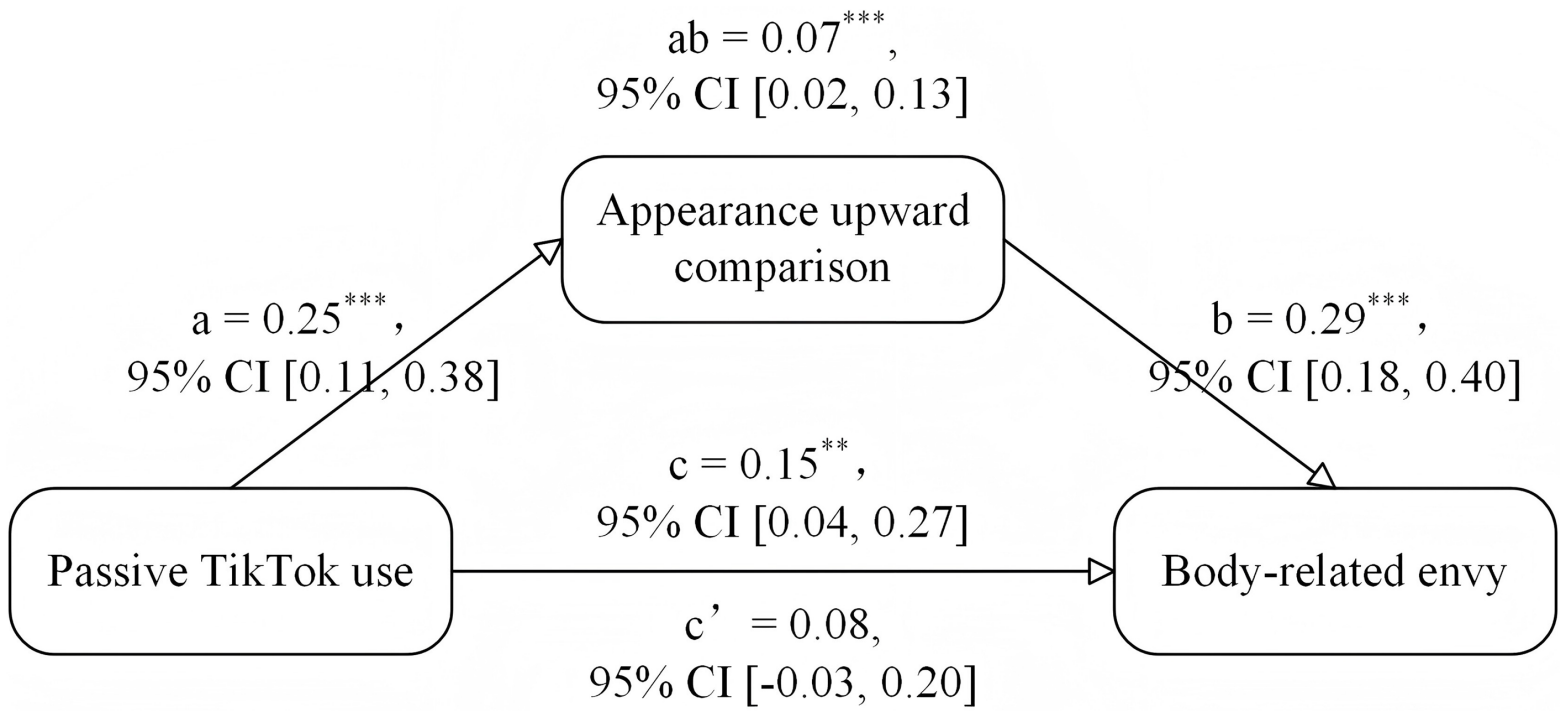
The mediation of appearance upward comparison between passive TikTok use and body-related envy in Study 2. ^**^p < 0.01, ^***^p < 0.001. Standardized coefficients were reported.

Based on the finding that passive TikTok use had a significant prediction effect on body-related envy, we applied the Macro Process (iterating 10,000 times) developed by Hayes (2013) to further test the possibly mediating role of appearance upward comparison between passive TikTok use and body-related envy. Age, BMI, monthly income, dispositional envy, and global self-esteem were included in the model as control variables. All variables were standardized before they were entered into the model. The results showed that the overall model was significant, *F*(7, 192) = 21.80, *R*^2^ = 0.44, *p* < 0.001. The indirect effect between passive TikTok use and body-related envy was significant, effect size = 0.07, 95% CI (0.02, 0.13), but the direct effect between them was not significant, effect size = 0.08, *t* = 1.46, *p* = 0.15, 95% CI (−0.03, 0.20). As shown in Figure 3, passive TikTok use significantly and positively predicted appearance upward comparison, *β* = 0.25, *p* < 0.001, 95% CI (0.11, 0.38), and appearance upward comparison further positively predicted body-related envy, *β* = 0.29, *p* < 0.001, 95% CI (0.18, 0.40). When the indirect effect of appearance upward comparison was taken into account, the prediction effect of passive TikTok use on body-related envy was not significant, *β* = 0.08, *p* = 0.15, 95% CI (−0.03, 0.20). The above results suggested that appearance upward comparison fully mediated the relationship between passive TikTok use and body-related envy.

### 6.3 Discussion

By conducting a questionnaire survey, Study 2 examined the relationship between active/passive TikTok use and body-related envy, and the possible mediating role of appearance upward comparison. The results of the hierarchical regressions showed that passive TikTok use had a significantly positive prediction on body-related envy, while active TikTok use did not have a significant prediction on body-related envy, thus providing evidence for H2a and H2b. Further analysis showed that appearance upward comparison played a fully mediating role between passive TikTok use and body-related envy, thus providing evidence for H2c.

Past research demonstrates that envy motivates women to take various measures to enhance their physical attractiveness, such as taking appearance-oriented exercise (Pila et al., 2014), using diet pills (Arnocky et al., 2016), and pursuing cosmetic surgery (Arnocky et al., 2016; Nabi and Keblusek, 2014). In recent years, the number of young women undergoing cosmetic surgery in China has been growing significantly (Sun, 2021). Given that, in the following Study 3, we aimed to explore the downstream consequences of female body-related envy—whether passive TikTok use further increases women’s cosmetic surgery consideration *via* the mediation of appearance upward comparison.

## 7 Study 3

The primary goal of Study 3 was to examine whether passive TikTok use would increase women’s cosmetic surgery consideration *via* the mediation of body-related envy. To achieve this goal, we again primed participants’ TikTok use state as in Study 2, and then measured their body-related envy and cosmetic surgery consideration. Independent-samples *t* tests were conducted to examine the possible between-groups differences in body-related envy and cosmetic surgery consideration. Based on this, the bootstrapping method was used to assess the mediating role of body-related envy between the experimental condition and cosmetic surgery consideration.

### 7.1 Methods

#### 7.1.1 Participants

Participants in Study 3 were recruited from the Credamo platform. Only women were allowed to take part in the study. The sample size of the study was determined by using the same method as that of Study 2. As a result, a total of 200 women were included in the final data analysis. Their average age was 32.03 years old (*SD* = 7.86), ranging from 18.08 to 57.00 years old. Their average BMI was 20.08 (*SD* = 2.42), ranging from 14.34 to 27.81. A total of 180 participants lived in the city, and 20 participants lived in the country. A total of 184 participants were of Han nationality, and others were from national minorities.

#### 7.1.2 Materials and measures

##### 7.1.2.1 Experimental manipulation

Considering that the priming materials in Study 1 had been validated in prior research (Yan et al., 2025) and that the samples of Study 1b and Study 3 were independent of each other, we reused the materials from Study 1b to implement the social media use manipulation in Study 3. This approach has been documented in prior social psychology research (e.g., Cheng et al., 2024a,b). In the TikTok use priming condition, participants were presented with a TikTok link that contained textual introductions to yoga pants and 10 pictures of women wearing yoga pants. In the control condition, participants were presented with a PDF document that contained textual introductions to yoga pants and 10 pictures of yoga pants. To reinforce the experimental manipulation, we used bold font to remind participants that they should not only pay attention to the text but also pay attention to the pictures.

##### 7.1.2.2 Body-related envy

The measure of body-related envy was identical to that of Study 1b. That is, participants needed to report to what extent they felt the following emotions on the 5-point scale (1 = *never*, 5 = *extremely*): resentful, longing for, jealous, and covetous. Body-related envy was assessed by summing the scores of the four emotions (Pila, 2013). The Cronbach’s *α* coefficient of the scale was 0.84.

##### 7.1.2.3 Cosmetic surgery consideration

The five-item consideration subscale of the Acceptance of Cosmetic Surgery Scale, developed by Henderson-King and Henderson-King (2005), was used to assess the extent to which participants would consider having cosmetic surgery to alter physical appearance. For each item, participants needed to provide their agreement on the 5-point scale (1 = *strongly disagree*, 5 = *strongly agree*). An example item was “in the future, I could end up having some kind of cosmetic surgery.” The score of cosmetic surgery consideration was calculated by averaging the scores of all items, with higher scores indicating more cosmetic surgery consideration. The Acceptance of Cosmetic Surgery Scale has shown satisfactory reliability and validity among Chinese samples (Jackson and Chen, 2015; Sun, 2021). In this study, the Cronbach’s α coefficient of the scale was 0.93.

##### 7.1.2.4 Control variables

As in Study 2, to control for possible confounding effects, we asked participants to report their dispositional envy and global self-esteem (Rosenberg, 1965; Tandoc and Goh, 2023). Additionally, we asked participants to report their age, height, weight, residence (country/city), nationality, and family’s monthly income.

#### 7.1.3 Procedure

Study 3 was conducted on the Credamo platform. Before the formal study, participants needed to sign the informed consent. Then, half of the participants were assigned to the passive TikTok use priming condition, and the other half of the participants were assigned to the control condition. Following the experimental manipulation, participants reported their body-related envy and cosmetic surgery consideration “at this moment.” Participants also reported dispositional envy and global self-esteem. Finally, participants reported demographic information and completed the experimental manipulation effectiveness check. Finally, participants were presented with a passage explaining that the study aimed to examine the influence of TikTok use on body-related envy and cosmetic surgery consideration. Those interested in further details could optionally provide their email address to receive additional information about the experimental design. Each participant would receive 5 YUAN (approximately 0.8 USD) as compensation for their participation.

### 7.2 Results

#### 7.2.1 The check of the manipulation effectiveness

Data analysis was performed using SPSS 26.0. As in Study 1b, the priming effectiveness of TikTok use was checked by asking participants to what extent the presented materials highlighted appearance-relevant content (1 = *not at all*, 7 = *strongly*). An independent-samples *t* test showed that the presented materials in the TikTok use priming condition (*M* = 6.24, *SD* = 0.92) were considered to more strongly highlight appearance-relevant content than those in the control condition (*M* = 3.21, *SD* = 1.86), *t*(198) = 14.59, *p* < 0.001, *d* = 2.06, suggesting the effectiveness of the experimental manipulation.

#### 7.2.2 Body-related envy and cosmetic surgery consideration between the two conditions

**Figure 4:**
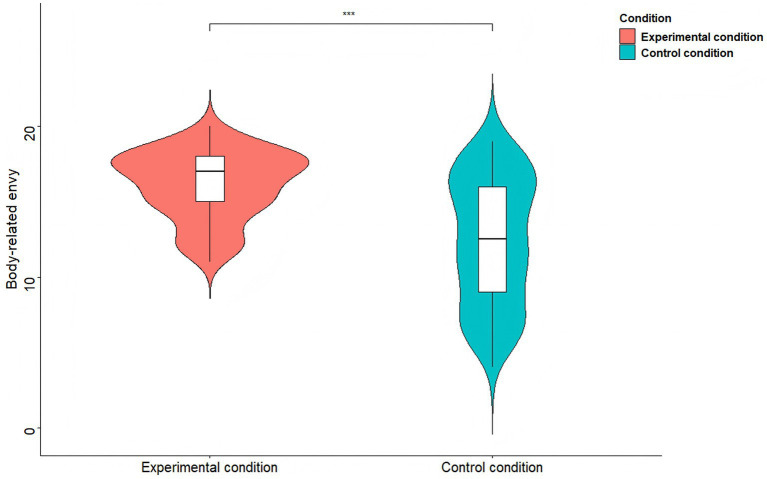
Body-related envy comparisons between the two conditions of Study 3. ^***^p < 0.001.

**Figure 5:**
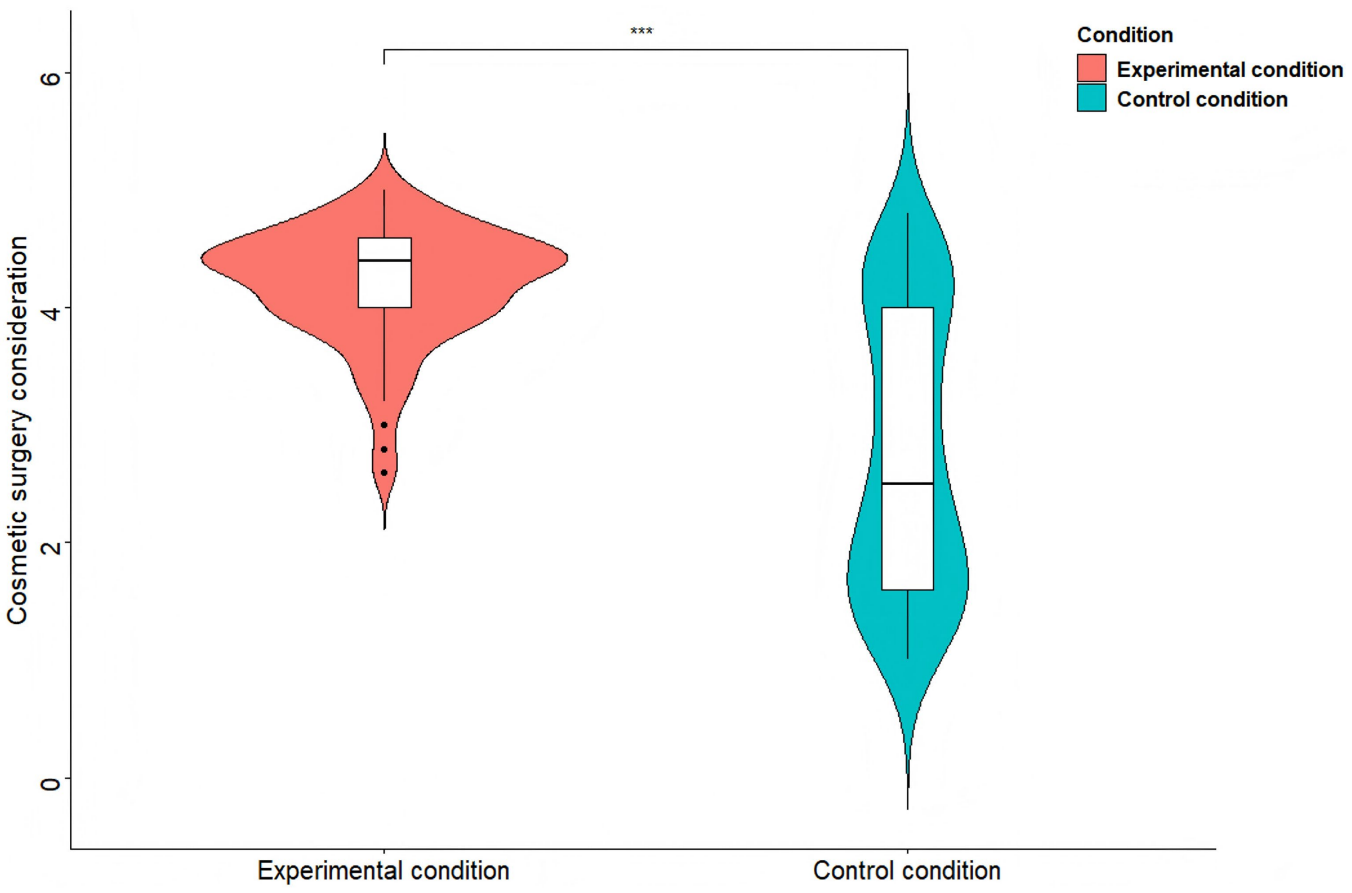
Cosmetic surgery consideration comparisons between the two conditions of Study 3. ^***^p < 0.001.

To examine whether experimental manipulation affects female body-related envy and cosmetic surgery consideration, two independent-samples *t*-tests were separately conducted. The results showed that participants in the TikTok use priming condition (*M* = 16.11, *SD* = 2.25) displayed greater body-related envy than those in the control condition (*M* = 12.39, *SD* = 4.18), *t*(198) = 7.85, *p* < 0.001, *d* = 1.11 (see Figure 4). Similarly, participants in the TikTok use priming condition (*M* = 4.19, *SD* = 0.50) reported stronger cosmetic surgery consideration than those in the control condition (*M* = 2.75, *SD* = 1.20), *t*(198) = 11.12, *p* < 0.001, *d* = 1.57 (see Figure 5).

#### 7.2.3 The mediating role of body-related envy

**Figure 6:**
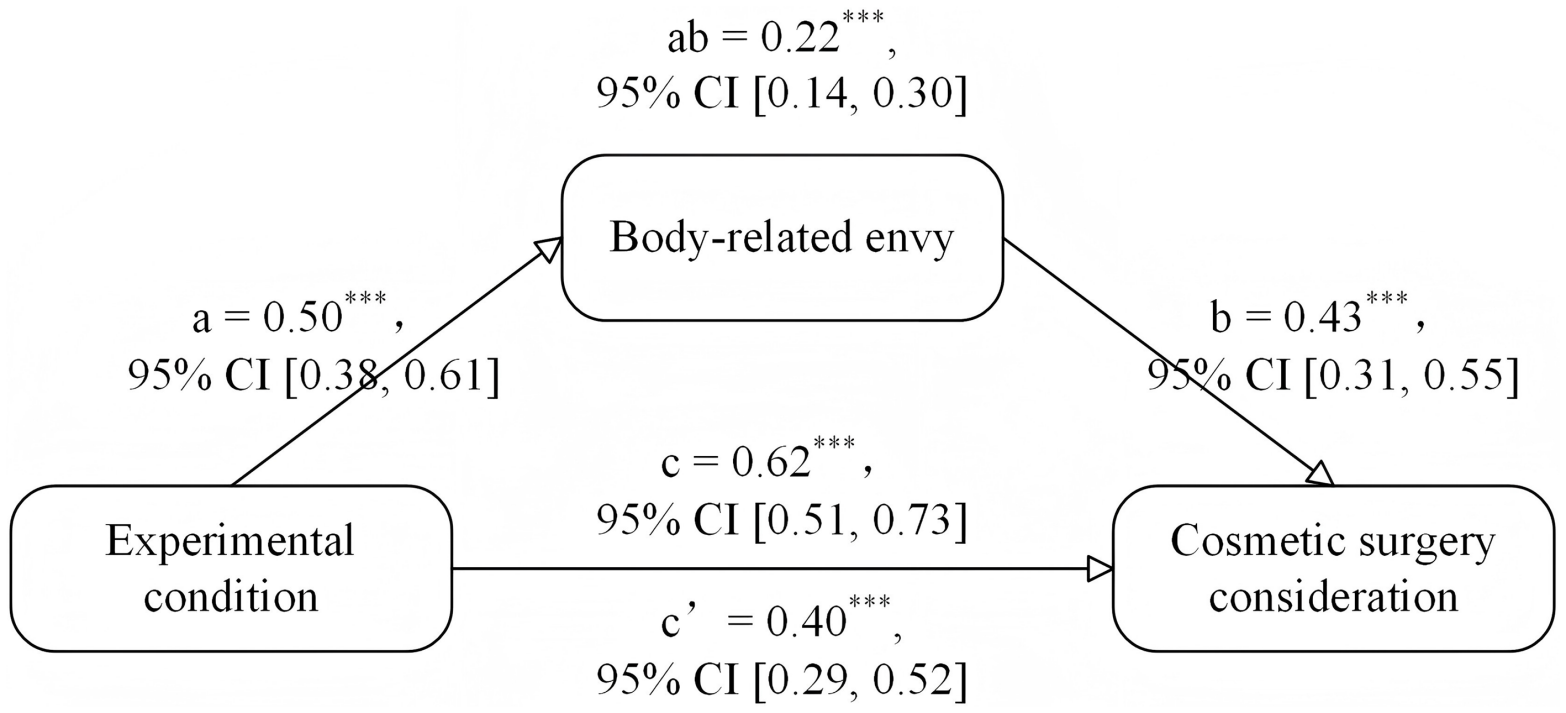
The mediation of body-related envy between the experimental condition and cosmetic surgery consideration. ^***^p < 0.001. The experimental condition was coded as a dummy variable (1 = TikTok use priming condition, 0 = control condition). Standardized coefficients were reported.

Considering that the experimental condition produced significant influences on body-related envy and cosmetic surgery consideration, the PROCESS macro (iterating 10,000 times) developed by Hayes (2013) was used to assess the mediating role of body-related envy between the experimental condition (1 = TikTok use priming condition, 0 = control condition) and cosmetic surgery consideration. As in Study 2, age, BMI, monthly income, dispositional envy, and global self-esteem were included in the model as control variables. All variables were standardized before they were entered into the equation. The overall mediation model was significant, *F*(7, 192) = 30.21, *R*^2^ = 0.52, *p* < 0.001. The indirect effect between experimental condition and cosmetic surgery consideration was significant, effect size = 0.21, 95% CI (0.14, 0.30). When the indirect effect was taken into account, the direct effect between experimental condition and cosmetic surgery consideration remained significant, effect size = 0.40, *t* = 6.87, *p* < 0.001, 95% CI (0.29, 0.52). As shown in Figure 6, the experimental condition had a significant prediction effect on body-related envy, *β* = 0.50, *p* < 0.001, 95% CI (0.38, 0.61), and body-related envy further had a significant prediction on cosmetic surgery consideration, *β* = 0.43, *p* < 0.001, 95% CI (0.31, 0.55). When the indirect effect of body-related envy was taken into account, the effect of experimental condition on cosmetic surgery remained significant, *β* = 0.40, *p* < 0.001, 95% CI (0.29, 0.52). The above results demonstrated that body-related envy partially accounted for the effect of experimental condition on women’s cosmetic surgery consideration.

### 7.3 Discussion

By conducting an online experiment, Study 3 found that participants in the TikTok use priming condition reported more body-related envy than those in the control condition. Importantly, the increased body-related envy led to the downstream consequences. That is, participants in the TikTok use priming condition reported more cosmetic surgery consideration than those in the control condition, and body-related envy partially mediated the effect of experimental condition on cosmetic surgery consideration, thus providing evidence for H3a and H3b. So far, all hypotheses proposed in the present research have received empirical support.

## 8 General discussion

By conducting four studies, the present research examined the effect and mechanism of TikTok use of female body-related envy and the subsequent downstream consequences. By conducting correlational and causal studies, Studies 1a and 1b found that TikTok use significantly increased female body-related envy. By classifying TikTok use into active and passive TikTok use, Study 2 found that passive TikTok use, rather than active TikTok use, was significantly related to female body-related envy. And appearance upward comparison fully mediated the relationship between passive TikTok use and female body-related envy. By conducting an online experiment, Study 3 further found that, partially *via* the mediating role of body-related envy, passive TikTok use significantly increased women’s cosmetic surgery consideration. The present research contributes to prior literature concerning social media use and female body-related envy.

### 8.1 Social media use and female body-related envy

The results of studies 1a and 1b suggested that TikTok use significantly increased female body-related envy, which was consistent with previous research concerning social media use and envy (Latif et al., 2021; Liu and Ma, 2020; Nabi and Keblusek, 2014; Tandoc and Goh, 2023). For example, a three-wave longitudinal study about Facebook use and depression showed that more Facebook use (Time 1) led to greater users’ envy (Time 2), which led to more depression (Time 3; Tandoc and Goh, 2023). In another study, based on the survey responses of 236 female students, Nabi and Keblusek (2014) found that social comparison to media figures significantly and positively correlated with participants’ envy. It should be pointed out that the present research extended prior research concerning social media and envy by first providing direct evidence that social media use can increase female body-related envy.

Social comparison theory assumes that people have an inner inclination to compare their opinions and abilities with those of others (Festinger, 1954). When an individual compares the self with someone better off (upward social comparison), envy will be triggered. Compared to offline settings, online settings allow individuals to present an “ideal self” in a more selective and controlled way (Gadgil et al., 2021), which enables individuals to experience more upward social comparisons in online settings than in offline settings (Appel et al., 2016). Moreover, some popular social media platforms—such as Facebook or TikTok—are highly visual (Tandoc and Goh, 2023). As a result, when using such social media platforms, users are especially susceptible to appearance upward comparison in those “highly visual” domains, such as physical appearance. That can explain why TikTok use increased female body-related envy in the present research.

### 8.2 Social media use, appearance upward comparison, and body-related envy

In Study 2, we found that active TikTok use did not significantly predict female body-related envy, while passive TikTok use significantly and positively predicted female body-related envy. Researchers have documented some activities relevant to active social media use, such as sharing life experiences, creating text or video content, and responding frequently to other users (Escobar-Viera et al., 2018; Montague and Xu, 2012). These activities can contribute to interpersonal connections among users and make them perceive social support (Clark et al., 2018). Social support has been identified as a protective factor for emotional distress (Thorisdottir et al., 2019). For example, with 910 adolescents as participants, Frison and Eggermont (2016) found that those girls who actively used Facebook, perceived online social support from active Facebook use, and perceived online social support further negatively predicted girls’ depressed mood. In addition, as we have documented in the Literature Review section, although a large body of research reveals the detrimental effect of social media use on self-esteem (Alfasi, 2019; Sun et al., 2020; Vogel et al., 2014), Han and Yang (2023) found that when individuals used social media with high social support perception, perceived social support counteracted the negative influences caused by upward social comparison, thus benefiting self-esteem. Based on these findings, we speculated that, maybe due to perceiving social support from active TikTok use, we did not observe a significant prediction effect of active TikTok use on female body-related envy.

In Study 2, we also found that appearance upward comparison mediated the relationship between passive TikTok use and female body-related envy. In contrast to active social media use, passive social media use implies that “users are not engaging with others and forging and maintaining social connections” (Quiroz and Mickelson, 2021). In this case, social media use actually cannot fulfill users’ needs for acceptance and belonging; users thus fail to perceive social support from such activities (Clark et al., 2018). Moreover, due to a lack of enough interpersonal connections, users with passive social media use often lack information about others’ real lives on social media (Clark et al., 2018). As a result, they may mistakenly perceive that others have better lives than themselves, thus experiencing upward social comparison. With respect to the present research, for those female users with passive TikTok use, they may mistakenly perceive that others on the platform display greater physical attractiveness than themselves, resulting in generating an appearance upward comparison. Appearance upward comparison was further positively associated with body-related envy.

### 8.3 Social media use, body-related envy, and cosmetic surgery consideration

The results of Study 3 showed that, *via* the mediating role of body-related envy, passive TikTok use significantly increased women’s cosmetic surgery consideration. Past research has well established the relationship between social media use and women’s cosmetic surgery consideration. For instance, with 238 young women as participants, Conboy and Mingoia (2024) adopted a questionnaire survey to examine the relationship among social networking site (SNS) use, self-compassion, and attitudes toward cosmetic surgery. The results showed that SNS use was significantly and positively correlated with women’s positive attitudes toward cosmetic surgery. As a common social media activity, selfie editing was found to have a positive correlation with cosmetic surgery consideration (Sun, 2021; Tao et al., 2024). Extending prior research, the present research first revealed that body-related envy mediated the effect of passive social media use on women’s cosmetic surgery consideration. As we have explained, passive social media use is more likely to make users experience upward social comparison, and cosmetic surgery can be regarded as a compensation strategy for those unfavorable social comparisons in the domain of physical appearance (Arnocky et al., 2016). By pursuing cosmetic surgery, women can exert some control over how they are viewed and treated by others, which will increase the probability of winning intrasexual competition (Arnocky et al., 2016, 2023; Calogero et al., 2014). Researchers have pointed out that cosmetic surgery can make women feel more satisfied with their appearance; they thus feel higher self-esteem and more confidence (Mironica et al., 2024).

It should be noted that, in addition to body-related envy, there are other possible mediators between passive TikTok use and women’s cosmetic surgery consideration. Prior literature suggests that social media use increases women’s appearance anxiety (Monro and Huon, 2005; Yao et al., 2024), and appearance anxiety is an important predictor of cosmetic surgery considerations (Ayar et al., 2025; Önalan et al., 2021). Thus, appearance anxiety may also mediate the effect of passive TikTok use on cosmetic surgery consideration in the present research. Body dissatisfaction is also a possible mediator between TikTok use and cosmetic surgery consideration. With 884 Chinese adolescents as participants, Wang et al. (2023) revealed that investment in others’ selfies was positively correlated with comparisons and general attractiveness internalization, which further positively predicted facial dissatisfaction. And facial dissatisfaction was finally positively related to adolescents’ cosmetic surgery consideration. In addition to mediating factors, potential moderators in the relationship between TikTok use and cosmetic surgery consideration also deserve our further attention. Recently, Wang et al. (2025) pointed out that individuals in collectivist cultures (e.g., China) are more likely to experience social comparison pressure. They found that there was a stronger link between appearance comparisons and negative body image among those participants experiencing greater sociocultural pressure. Thus, future research may need to examine how cultural background or perceived sociocultural pressure moderates the observed patterns of results.

### 8.4 Limitations and future work

There are several limitations in the present work. First, considering that TikTok is a video-based social media platform widely used in China, we must be cautious in extrapolating these results to other media platforms. Prior research suggests that video/picture-based social media platforms, to a greater extent, highlight appearance-relevant content in comparison to those text-based social media platforms (Fardouly et al., 2015; Mironica et al., 2024; Tiggemann et al., 2018). Thus, compared to text-based social media platforms, using video/picture-based social media should be more likely to induce female body-related envy. In other words, the effect of social media use on female body-related envy may vary with social media type, and it remains unclear the degree to which the observed effect of TikTok use on female body-related envy can be generalized to other social media platforms. Methodologically, the data in several studies were collected *via* self-report questionnaires, which makes it difficult to rule out the influence of social desirability bias. This limitation may also, to some extent, affect the generalizability of our findings.

Second, compared to older women, younger women display more body image concerns (Pruis and Janowsky, 2010; Tiggemann, 2004). As a consequence, younger women should be more likely to show body-related envy than older women when they are exposed to appearance-relevant content on social media. However, the present research did not examine whether and to what extent age would moderate the effect of TikTok use on female body-related envy, which more or less threatens the generalization of our findings. Additionally, body image concerns are prevalent in both men and women, though their manifestations differ—women typically prioritize thinness, whereas men emphasize leanness and muscularity (Cheng et al., 2024a,b; Dunstan et al., 2017). Future research should examine the generalizability of our findings to male populations.

Third, the present research examined the effect and the underlying mechanism of passive TikTok use on female cosmetic surgery consideration. The results showed that passive TikTok use increased female cosmetic surgery consideration partially *via* the mediating role of body-related envy. As we have discussed, in addition to body-related envy, there are other possible mediators between passive TikTok use and female cosmetic surgery consideration, such as body dissatisfaction, appearance anxiety, and body shame. Additionally, according to our previous reasoning, active and passive social media use both should increase female cosmetic surgery consideration, but different mechanisms should underlie the effects of active and passive social media use on female cosmetic surgery consideration. Given the above considerations, more work should be conducted to comprehensively investigate how active and passive social media use increases female cosmetic surgery consideration.

Finally, while the present research reveals that social media use can produce negative influences on women’s body image—manifested as heightened body-related envy and cosmetic surgery consideration, it does not further explore potential strategies to mitigate these adverse effects. Despite this, prior research may offer some feasible intervention strategies. For example, social media literacy interventions—programs designed to enhance users’ critical thinking about media content and foster skepticism toward platform algorithms have been shown to mitigate the negative effects of social media use on women’s body image (McLean et al., 2017; Tamplin et al., 2018). As of 2023, TikTok (Douyin) has surpassed 400 million daily active users in China, with the 18–24 age group comprising roughly 30% of this total (QuestMobile, 2024). Excessive TikTok use may drive some young women (including college students) to engage in frequent self-scrutiny and even pursue high-risk cosmetic surgeries. Given that, future research could examine the extent to which media literacy interventions mitigate the effects of TikTok use on female body-related envy and cosmetic surgery consideration.

## 9 Conclusion

The present research examined the effect and mechanism of TikTok use on female body-related envy and the subsequent downstream consequences. Data analysis results suggested that compared to active TikTok use, passive TikTok use was more likely to increase female body-related envy *via* the mediating role of appearance upward comparison. Additionally, *via* the mediating role of body-related envy, passive TikTok use increased women’s cosmetic surgery consideration. The present research contributes to our understanding of how social media use increases women’s body image concerns and appearance enhancement intentions.

## Data Availability

The datasets presented in this study can be found in online repositories. The name of the repository/repositories and accession number(s) can be found below: The datasets of the present research can be found on OSF (https://osf.io/2dz6k?view_only=d9eb3830986a40a5b68c09f066b42958).

## Footnotes

^1^ www.credamo.com

^2^ https://osf.io/fnz95?view_only=d9eb3830986a40a5b68c09f066b42958
